# Does hope mediate the relationship between parent’s resolution of their child’s autism diagnosis and parental stress

**DOI:** 10.3389/fpsyg.2024.1443707

**Published:** 2024-09-04

**Authors:** Vrinda V. Naicker, Darren Hedley, Simon M. Bury

**Affiliations:** Olga Tennison Autism Research Centre, School of Psychology and Public Health, La Trobe University, Melbourne, VIC, Australia

**Keywords:** resolution, autism spectrum disorder, diagnosis, hope, parent–child relationship, parental wellbeing, acceptance

## Abstract

**Introduction:**

Resolution of a child’s diagnosis, the process of accepting and adjusting to the reality of a child’s significant diagnosis, has been often associated with decreased parental stress. Hope, a potential buffer against psychological distress, has been suggested as a potential explanation for this relationship. However, the mediating role of hope in the relationship between resolution of diagnosis and parental stress has not been explored.

**Methods:**

This study aimed to examine whether four types of hope (child, parental, societal, denial of diagnosis) mediated the relationship between resolution to an autism diagnosis and reduced parental stress. Participants included 73 parents (*M_age_ =* 43.22, *SD* = 7.69, female 97.3%) of autistic children (*M_age_* = 11.15, *SD* = 4.56, male = 67.1%).

**Results:**

Resolution to diagnosis was negatively and significantly correlated with resolution to diagnosis, as well as child, parental and societal hope. These three hopes were also significantly and negatively correlated with parental stress. Importantly, when controlling for level of support and autism awareness, parental hope mediated the relationship between resolution to diagnosis and parental stress. Denial of diagnosis was not correlated with resolution or parental stress but did have significant but weak associate with the other hopes.

**Discussion:**

These findings suggest that hope based on parent’s abilities to support their child and be supported themselves play an important role in parental stress once parents are more resolved to their child’s diagnosis. Supporting parents to manage factors associated with supporting their child’s needs, may benefit parents of autistic children.

## 1 Introduction

Receiving an autism spectrum disorder (hereafter “autism”; American Psychiatric Association, 2013) diagnosis for a child can be a stressful experience for parents (Poslawsky et al., 2014). Parents’ ability to adjust their expectations of parenthood from pre-diagnosis to post-diagnosis may contribute to this increased stress (Milshtein et al., 2010). This adjustment process, where parents come to terms with the implications of the diagnosis, is known as resolution to diagnosis (Marvin and Pianta, 1996; hereafter “resolution”). Research shows that parents who achieve higher resolution regarding significant diagnoses for their children (e.g., Cerebral Palsy, autism, and psychiatric disorders) often experience positive outcomes, such as reduced parental stress (Sher-Censor and Shahar-Lahav, 2022; Lord et al., 2008). However, less is known about the mechanisms behind why resolution may lead to reduced stress. Increased hope, which acts as a protective factor against psychological distress (Mednick et al., 2007; Ogston et al., 2011), has been suggested as a potential explanation. Despite this, the relationship between resolution and hope has been inconsistent, possibly due to how hope is conceptualized in these studies. We propose that a goal-focused hope, grounded in reality (Bury et al., 2016), may better explain the relationship between resolution and reduced parental stress in parents of autistic children.

### 1.1 Autism and parental stress

Autism is a neurodevelopmental condition characterized by differences in social communication and interaction skills, as well as restricted, repetitive patterns of behavior (American Psychiatric Association, 2013). These differences can impact autistic children’s inclusion in daily activities, such as school, leading to varying levels of required support in these domains. Diagnosing autism in childhood allows parents the opportunity to provide additional behavioral or academic support during critical developmental years (Okoye et al., 2023). However, the diagnostic and support process can also cause significant distress for parents (Moh and Magiati, 2012).

Parents of children diagnosed with autism experience higher levels of stress compared to parents of typically developing children and those with other disabilities (Bonis, 2016; Dabrowska and Pisula, 2010; Smith et al., 2001). Stress may stem from the additional demands parenting an autistic child may present, such as managing behaviors that challenge, changes in the parent’s life (e.g., routines, relationships with friends and family), as well as difficulties finding and organizing autism services, or overcoming barriers or challenges with the education systems (Bonis, 2016, Hayes and Watson, 2013). Managing this stress is crucial, as it can impact parent–child relationships and the overall wellbeing of both the child and parent (Schieve et al., 2007). Increased stress can alter parenting behaviors, which may influence the child’s symptoms and behaviors (Eshraghi et al., 2022).

Behavioral characteristics, such as the level of challenging behaviors and support needs, are particularly associated with elevated stress levels (Dabrowska and Pisula, 2010; Estes et al., 2009; Pastor-Cerezuela et al., 2016; Yirmiya et al., 2015). Higher support needs place greater demands on parents (Krakovich et al., 2016). Given the impact of stress on the family dynamic, focusing on factors that reduce stress is essential for improving family functioning (Hayes and Watson, 2013).

### 1.2 Resolution to diagnosis

Resolution to diagnosis is suggested to help parents better manage this stressful time (Sheeran, et al., 1997). Marvin and Pianta (1996) describe resolution as a process of accepting a diagnosis in its entirety, understanding the implications of the diagnosis, and parents adjusting their internal representations and expectations of their child from pre-diagnosis to post-diagnosis. This process involves aligning prior internal representations with the new reality of having a child with different needs (Milshtein et al., 2010).

Derived from attachment theory, particularly secure attachment (Marvin and Pianta, 1996; Oppenheim et al., 2009), resolution involves parents being emotionally available to understand their child’s emotions and meet their caregiving needs (Bowlby, 1969; Allen et al., 1996). Sheeran et al. (1997) found that resolution affects intimate family relationships and broader social circles surrounding the family, making it important to identify factors that impact this process. Da Paz et al. (2018) report that the severity of a child’s symptoms is positively associated with resolution. Additionally, time since diagnosis and prior knowledge of the diagnosis and its impact are linked to increased resolution and reduced stress levels (Al-Oran and Al-Sagart, 2016).

Parents’ knowledge and uncertainty about the diagnosis significantly affect their understanding of autism, contributing to a sense of loss. A lack of knowledge can prevent parents from understanding their child and accessing appropriate support, while greater knowledge serves as a protective factor (Alsayyari, 2018; Heredia-Alvarado, 2017; Rabba et al., 2019). Achieving acceptance and resolution is associated with positive emotional responses and improved interactions with their child (Heredia-Alvarado, 2017; Hotez, 2016; Rabba et al., 2019). In contrast, negative emotions such as denial and blame elongate the resolution process (Ferguson and Vigil, 2019).

Resolved parents experience less guilt, blame, and shame regarding the autism diagnosis (Naicker et al., 2023; Da Paz et al., 2018; Heredia-Alvarado, 2017). Resolution acts as a protective factor, reducing feelings of shame and denial and enhancing parent wellbeing, attunement, and insightfulness in the parent–child relationship (Naicker et al., 2023). Therefore, understanding the factors that influence the resolution process is crucial.

#### 1.2.1 Resolution and stress

One of the common benefits of resolution is its association with lower stress levels among parents (Sheeran et al., 1997; Lord et al., 2008). A scoping review by Sher-Censor and Shahar-Lahav (2022) found unresolved narratives associated with higher parenting stress among 16 studies, including childhood diagnoses of autism, Phenylketonuria, down syndrome, cerebral palsy, Type 1 Diabetes, and psychiatric disorders (e.g., attention deficit hyperactivity disorder and mood disorder). This review found unresolved narratives up to a month after receiving the diagnosis were specifically related to higher stress. A review by Naicker et al. (2023) found that parents with higher resolution reported reduced psychological distress and depression, an overall higher capacity to cope with stress, higher levels of marital satisfaction, and seeking and accessing social support. Importantly, although parents of children diagnosed with autism experience higher levels of stress (Smith et al., 2001), resolution is still associated with lower stress in parents of children with autism (Sheeran et al., 1997). However, while the relationship between resolution and stress is established, explanations for why this relationship exists are less developed. One factor suggested to underpin this relationship is hope.

#### 1.2.2 Resolution and hope

While discussed as a factor of resolution, few studies discuss the direct link between resolution and hope. Some studies found that mentions of hope arise when examining parental experiences with diagnostic processes and raising a child (Rabbitte et al., 2017). Another study found hope to arise regarding the future of their child: the increasing independence of their child and hope surrounding support systems to facilitate their child’s needs (Benderix et al., 2006). However, there is inconsistent evidence regarding the nature of the relationships between hope and resolution (Sher-Censor and Shahar-Lahav, 2022). For example, while Lord et al. (2008) found no significant association between resolution and hope, Popp et al. (2015) found parents to have greater hope when resolved to their child’s Cancer diagnosis. Differences in outcomes may arise when considering theoretical considerations of hope.

### 1.3 Hope in uncertainty

Hope has traditionally been conceptualized in psychology as a positive expectancy construct (see Hope Theory; Snyder et al., 1991), in which a greater perceived likelihood of success results in higher levels of hopefulness. Theorized this way, hope has similar outcomes to other expectancy-based outcomes (e.g., self-efficacy and optimism), thus the unique nature of hope is unclear. Rather than an expectation of success, alternative approaches to hope show that hope arises in uncertainty (Bury et al., 2016; Miceli and Castelfranchi, 2010; Ogston et al., 2011), when individuals are less assured of success (Bury et al., 2019). Such conceptualizations are more in line with qualitative descriptions of hope, highlighting that hope as emotion is future-focused, and arises for uncertain or uncontrollable outcomes (Bruininks and Malle, 2005), outcomes not well captured in expectancy-based theories of hope. Hope in uncertainty does not suggest that people downplay or ignore the odds, rather for outcomes that are of significant importance to the individual, it is precisely the uncertainty of success that requires one to hope (Bury et al., 2016). Conceptualized thus, research shows hope to be distinct from expectancy measures (Bury et al., 2019) and associated with intention to act, even for outcomes outside one’s sole agency (Bury et al., 2020).

When receiving a significant diagnosis for their child, parent’s uncertainty about their child’s future and their ability to support their child’s needs suggest a good environment for hope. Research suggests that caregivers who receive a significant childhood diagnosis perceive greater uncertainty about the diagnosis and future outcomes for their child and parenting, which is associated with greater initial distress as well as additional hope (Mulligan et al., 2012; Sanders-Dewey et al., 2001). Studies have found hope arises during the diagnostic process with some parents reporting an initial loss of hope when receiving the diagnosis or regarding their initial expectations surrounding therapies, contrastingly gaining a sense of hope within the uncertainty of receiving the diagnosis often from surrounding supports (i.e., professionals, social circles; McPhilemy and Dillenburger, 2013; Reiff et al., 2017; Rabba et al., 2019). Singh (2016) found parents who receive their child’s diagnosis and are presented with initial expectations surrounding the condition, describe minimal hope for the future. However, these parents reported having to create hope, leading to opportunities to challenge boundaries placed on their child’s diagnosis.

In terms of resolution, the resolution includes parent’s expectations and internal representations of their child aligning with their child’s diagnosis, which promotes realistic, but uncertain, expectations for their child’s future (Sher-Censor and Shahar-Lahav, 2022). Dolev et al. (2016) found the process of refocusing attention and realistic expectations leads parents to have a greater understanding of appropriate parenting methods for their child, resulting in parents better responding to child cues. Thus, a hope grounded in possibility, not probability, likely presents a greater resource for parents at such times. Indeed, initial evidence in other domains suggests hope may be a good facilitator of goal-consistent action at such times (Bury et al., 2020).

#### 1.3.1 Stress and hope

Importantly, hope has also been suggested to be a protective factor against psychological distress (Mednick et al., 2007). Ong et al. (2006) found individuals with higher hope displayed reduced stress reactivity, effective emotional recovery, and the ability to keep negative emotions low. Receiving a child’s diagnosis can be strenuous on parent’s wellbeing, with parents reporting the need for hope during the diagnostic process (Nissenbaum et al., 2002). In children diagnosed with Phenylketonuria, Lord et al. (2008) found higher levels of personal hopefulness to be associated with lower levels of parenting stress, while Mednick et al. (2007) identified hope as a protective factor against psychological distress in mothers of children diagnosed with type 1 diabetes. However, little research discusses the relationship between stress and hope in the context of autism, even more so with hope as a mediating factor between stress and resolution. Additionally, the theoretical perspective of hope in this research is not clear, with parents describing hope as including optimism (Nissenbaum et al., 2002), an expectancy-based construct often confused for hope (Bury et al., 2019).

#### 1.3.2 Hope and receiving an autism diagnosis

Hope, then, is a positive future-focused emotion, arising from possible but uncertain goals (Bury et al., 2016; Miceli and Castelfranchi, 2010; Wenzel et al., 2017). To better understand what specific goals parents of children with an autism diagnosis have that may engender hope, we have reviewed the literature to identify specific goals of hope. We found that parents have reported different descriptions of specific hopes, which have been categorized into four different categories: hope for the child, parental hope, societal hope, and denial of diagnosis.

*Hope for the child* reflects realistic and positive goals for their child’s future and encompasses goals such as parents’ hopes to form meaningful relationships with their child, and future hopes for their child to live a good quality and emotionally fulfilling life (Benderix et al., 2006; McPhilemy and Dillenburger, 2013; Mulligan et al., 2012; Ogston et al., 2011; Reiff et al., 2017; Rabba et al., 2019; Singh, 2016; Blake, 2021). Given a focus on positive strength-based outcomes for the child, resolution seems important for these hopes to arise.

*Parental hope* reflects parents’ own hopes for themselves, specifically their ability to support their child financially and mentally and to be able to meet their child’s needs (Blake, 2021; Ekas et al., 2016; Mulligan et al., 2012; Ogston et al., 2011). Parental hope also represents parents’ hopes about being supported themselves.

*Societal hope* reflects parent’s hopes for the greater community coming to better understand and value autism (Ekas et al., 2016; Rabba et al., 2019). This hope is bigger than the individual and represents a broader hope of their child being recognized and supported by society for who they are.

*Denial of diagnosis* potentially represents more a denial than of a true hope based on reality (McPhilemy and Dillenburger, 2013; Mulligan et al., 2012; Reiff et al., 2017). Rather than a realistic representation of the future, hopes around the denial of diagnosis include wishing that the diagnosis was wrong, could be corrected, or that the child will still be able to meet the parent’s preconceived expectations. Given this hope does not reflect resolution to a diagnosis, we would not expect it to be associated with resolution of diagnosis, or to reduced stress.

### 1.4 The current study

**Figure 1:**
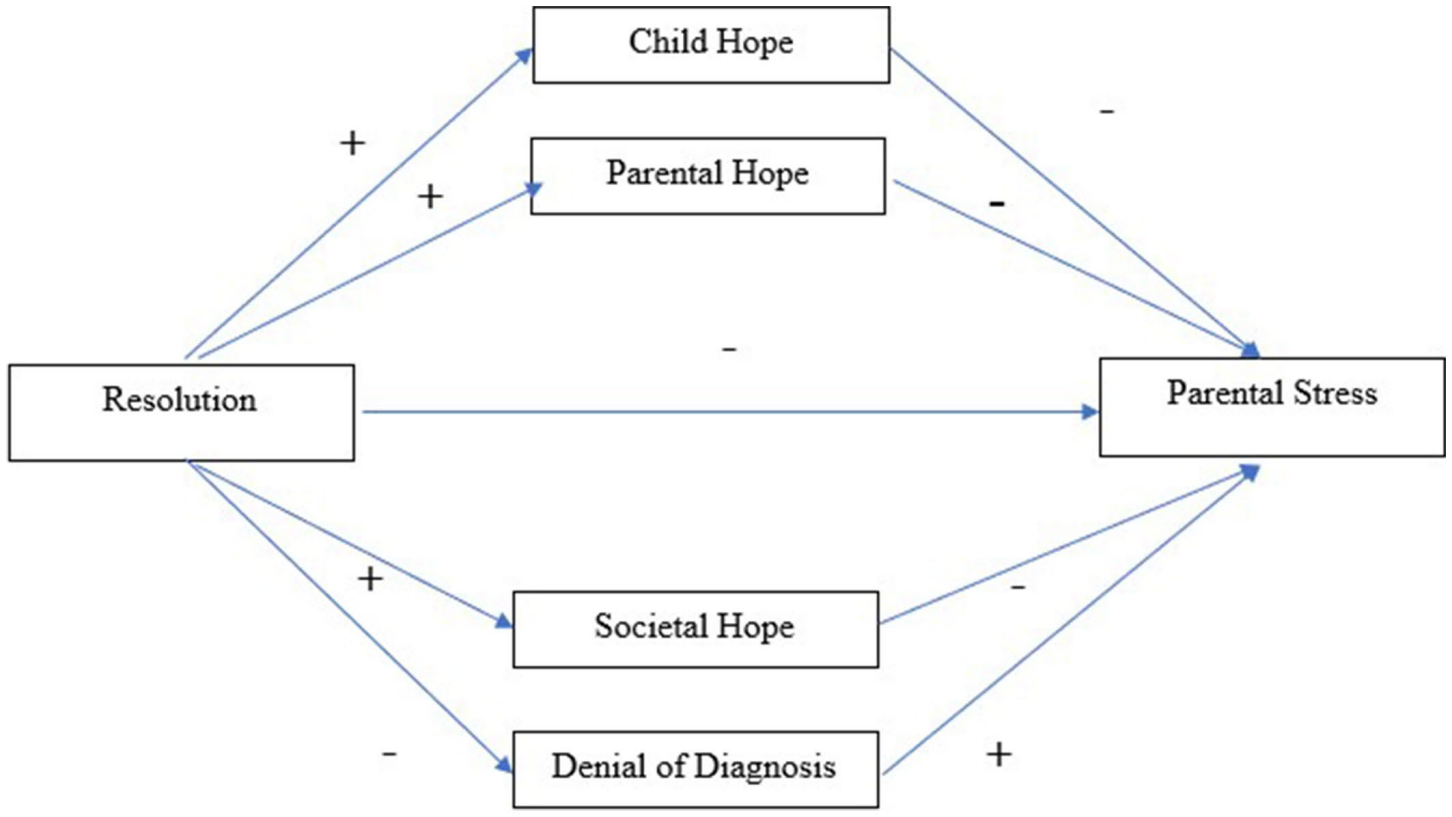
Predicted indirect path of resolution and parental stress via hope (Child, Parental, Societal, Denial of Diagnosis).

We investigated the relationship between resolution to diagnosis and parental stress in parents of children with an autism diagnosis. Specifically, we were interested in whether hope mediates the relationship between resolution and parental stress, and what type of hope best explains this relationship. We conducted a single mediation model with multiple hope mediators (Figure 1). We predicted there would be a negative relationship between resolution and parental stress (Hypothesis 1). Next, we predicted there would be an indirect effect of resolution to diagnosis and parental stress via child hope, parental hope, and social hope, whereby resolution will lead to greater hope, and in turn, lower parental stress (Hypotheses 2, 3, and 4). For hope that denies the diagnosis, we tentatively predict that resolution would be negatively associated with negative hope, and positively associated with parental stress.

## 2 Method

### 2.1 Participants

Participants were 73 parents of autistic children (*M_age_*  = 43.22, *SD* = 7.69, female = 97.3%, male = 2.7%) who completed the survey (see Table 1 for sample demographics). Participant’s children (*M_age_*  = 11.15, SD = 4.56, Male = 67.1%; Female = 32.9%) were all diagnosed with autism, with a mean diagnosis age of 5.5 (*SD* = 3.45). Most parents (75%) knew their child’s level of support (Level 1: 13.0%, Level 2: 66.7%, Level 3: 20.4%), with the remaining (25%) parents reporting the perceived level of severity of their child (Level 1: 21.1%, Level 2: 63.2%, Level 3: 15.8%).

**Table 1:** Parent demographic information, frequency, and percentages.

Demographic	Frequency	Percentage (%)
Parent gender		
Female	71	97.3
Male	2	2.7
Other	0	0
Ethnicity		
Australian	61	83.6
Indigenous Australian or Torress Strait Islander	1	1.4
Asian	1	1.4
Middle Eastern	3	4.2
European	5	6.8
Other	1	1.4
Marital status		
Married/Domestic partnership	57	78.1
Divorced/Separated	10	13.7
Single	6	8.2
Level of education		
Less than high school	1	1.4
High school Graduate	4	5.5
Vocational/Trade/Technical school	2	2.7
Diploma/Certificate	16	21.9
Bachelor’s degree	28	38.4
Postgraduate degree	22	30.1
Employment status		
Unemployed	21	28.9
Part-time	34	46.6
Casual	9	12.3
Full-time	9	12.3
Total annual household income		
Less than 40 k	12	16.4
40–60 k	5	6.8
60–80 k	4	5.5
80–100 k	13	17.8
100–120 k	8	11
More than 120 k	31	42.5
Number of children		
1	15	20.5
2	37	50.7
3	14	19.2
4 or more	6	8.2
Prior relationship with autism (multiple responses)		
My other child	25	34.2
My sibling	7	9.6
A family member of mine	27	37
Someone closely associated with	20	27.4
Acquaintance	26	35.6
Did not know anyone with autism	18	24.7

### 2.2 Measures

Participants first completed a demographic questionnaire collecting general information (age, gender, marital and employment status, household information, etc.), the parents’ own characteristics, information regarding the child’s diagnosis, prior knowledge, and prior relationships with individuals with autism. They were then presented with measures for the main variables in the study.

#### 2.2.1 Resolution

The Reaction to Diagnosis Questionnaire (RDQ) (Sher-Censor et al., 2020) is a new self-report measure assessing parental resolution of their child’s diagnosis. The RDQ is a 42-item, 5-point Likert scale (*1 = Strongly Disagree, 5 = Strongly Agree*), showing good internal consistency across two sample sizes (Sher-Censor et al., 2017). This study purposefully removed an item from the RDQ due to a similar hope formulation to items in the denial of hope (Item 38 “I hope that my child’s condition will improve with time”), while another item was excluded by error (Item 29). Higher scores indicated higher resolved parents (McDonald’s *ω* = 1.20).

#### 2.2.2 Parental stress

The Parental Stress Scale (PSS) is a widely used standardized scale measuring parental stress (Berry and Jones, 1995). It includes 18 items and a 5-point Likert scale (*1 = Strongly Disagree, 5 = Strongly Agree*). It encompasses both the demands and fulfilling aspects of parenting, showing adequate reliability and good test–retest reliability and strengths as a cross-cultural measure, presenting possible face validity (Louie et al., 2017). Scores were then averaged with higher scores indicating greater stress levels (McDonald’s *ω* = 1.06).

#### 2.2.3 Hope

Hope was measured with a new measure targeting four hope-based goals, based on Wenzel et al. (2017). In total, 19 hope statements were created based on prior research (see Introduction section). These studies revealed that parents experienced different sub-sets of hope regarding their children. Thus, the statements examined were categorized into four hope categories. *Child hopes* (McDonald’s *ω* = 0.96) included eight statements of hope for their child (“I am hopeful that my child will have a good quality of life”). *Parental hope* (*ω* = 0.89) included five statements about the parent’s ability to meet the needs of their child (“I am hopeful that I can be the parent that my child needs me to be”). Societal hope (*ω* = 0.97) included three items reflecting greater acceptance of autism in society (“I am hopeful that society will see the strengths associated with autistic people”). Denial of diagnosis (*ω* = 0.75) included three items that reflected a denial or hope that the diagnosis was wrong (“I am hopeful that my child’s diagnosis was a mistake”). All items were measured with a 7-point Likert scale (*1 = Strongly Disagree, 7 = Strongly Agree*), with a higher mean indicating higher levels of hope in that specific category.

#### 2.2.4 Covariates

We measured four variables to be potential covariates, based on their association with resolution or parental stress in the literature. *Autism Knowledge:* The Autism Awareness Survey (AAS) (Gillespie-Lynch et al., 2015) was used to assess the accuracy of parent’s knowledge of autism. It includes 13 items measured on a 5-point Likert scale (*1 = Strongly Disagree, 5 = Strongly Agree*) with higher scores indicating a higher level of knowledge about autism y (*α* = 0.69). *Time since diagnosis* was calculated by subtracting the date of diagnosis provided by parents in the demographic survey from the date the survey was taken. *Prior relationship with autism*: parents were asked if and whom they knew who also had an autism diagnosis, this was then coded into a numerical value (0 = no prior relationship, 1 = prior relationship). *The level of support* was calculated through demographic questions asking parents about known or perceived level *(Level 1 = Requires support, Level 3 = Requires very substantial support)* of their child’s support level. Participants only reported perceived level if they did not know their child’s support level, with perceived level used in the absence of the actual level of required support.

### 2.3 Procedure

This study was granted ethics approval by the La Trobe University Human Research Ethics Committee (Approval number: HEC22124). Participants were recruited via social media of the Olga Tennison Autism Research Centre (OTARC), particularly targeting autism-specific forums, groups, and organizations. Inclusion criteria for participants required the parents of autistic children to be 18 years of age and over. To ensure greater control for broad societal and cultural differences, participants were required to be Australian citizens or permanent residents. Interested individuals were led to the online survey hosted via REDcap (Harris et al., 2019) via the link provided on all advertisement materials. Upon completion of the survey, participants entered a prize draw to win one of ten $50 supermarket gift vouchers.

### 2.4 Analytic plan

Only 0.29% of the variable data was missing, with Little’s MCAR test Chi-Square (835) = 138.27, *p* = 1.000, indicating that data were missing at random. Missing data were imputed from the mean of adjacent variables to ensure maximum sample size. One participant only responded to 15% of items for the Autism Awareness Scale, these data could not be imputed so were marked as missing for this measure.

Data were collated and prepared using Statistical Package for Social Scientists, Version 28.0 (SPSS). To test the internal validity of identified factors, McDonald’s Omega was used. Correlation was run using SPSS with 1,000 Bootstrapped confidence intervals indicating significance, including all key variables and the four potential covariates. Mediation analysis was conducted using Model 4 of the PROCESS Macro in SPSS (Hayes, 2018) with 5,000 bootstrapped samples, with covariates that correlated significantly with model variables included as covariates.

## 3 Results

### 3.1 Correlation between resolution and parental stress

Pearson correlations were run between all variables, including covariates (Table 2). Resolution had significant positive moderate to strong correlations with child hope, parental hope, and societal hope. Stress was negatively and significantly correlated with child, parental, and societal hope, but there was no significant correlation between stress and hope that denies the diagnosis. Child, parental, and societal hopes were all significantly and positively correlated, with parental and societal hopes strongly and significantly positively correlated. Denial of diagnosis was significantly positively, yet weakly, correlated with the other hope items. Level of support was negatively correlated with denial of diagnosis, as was autism awareness. Prior relationship and times since diagnosis did not correlate with any other variable.

**Table 2:** Correlations between variables of interest with bias corrected accelerated bootstrapped confidence intervals (95%).

	Variable	Mean	SD	1	2	3	4	5	6	7	8	9
1.	Resolution	3.80	0.53	1								
2.	Child hope	5.67	1.37	**0.59 [0.35, 0.75]**	1							
3.	Parental hope	5.87	1.31	**0.53 [0.28, 0.71]**	**0.64 [0.55, 0.88]**	1						
4.	Societal hope	6.02	1.56	**0.45 [0.19, 0.65]**	**0.62 [0.38, 0.79]**	**0.78 [0.62, 0.88]**	1					
5.	Denial of diagnosis	2.20	1.23	−0.15 [−0.39, 0.09]	**0.27 [0.09, 0.42]**	**0.26 [0.08, 0.44]**	**0.24 [0.04, 0.41]**	1				
6.	Parental stress	2.65	0.67	**−0.68 [−0.80, −0.51]**	**−0.55 [−0.71, −0.30]**	**−0.58 [−0.74, −0.35]**	**−0.51 [−0.67, −0.28]**	−0.12 [−0.32, 0.11]	1			
7.	Level of support	2.04	0.59	−0.13 [−0.42, 0.13]	−0.14 [−0.43, 0.12]	−0.09 [−0.36, 0.16]	0.004 [−0.25, 0.22]	**−0.23 [−0.41, −0.05]**	0.12 [−0.15, 0.37]	1		
8.	Time since diagnosis	5.53	4.36	−0.03 [−0.26, 0.19]	−0.04 [−0.25,0.19]	0.07 [−0.15, 0.28]	0.08 [−0.18, 0.29]	−0.10 [−0.32, 0.14]	0.06 [−0.15, 0.26]	0.10 [−0.15, 0.35]	1	
9.	Prior relationship	–	–	0.20 [−0.01, 0.43]	0.06 [−0.18, 0.32]	0.04 [−0.21, 0.29]	0.02 [−0.19, 0.27]	−0.12 [−0.38, 0.09]	−0.21 [−0.42, 0.01]	−0.01 [−0.25, 0.21]	0.03 [−0.22, 0.29]	1
10.	Autism Awareness (knowledge) (*n* = 72)	4.39	0.37	**0.27 [0.02, 0.50]**	−0.09 [−0.31, 0.17]	−0.10 [−0.31, 0.17]	−0.07 [−0.27, 0.19]	**−0.49 [−0.67, −0.26]**	0.03 [−0.22, 0.26]	−0.07 [−0.27, 0.15]	0.06 [−0.19, 0.30]	0.13 [−0.13, 0.39]

Bolded data indicate significant correlations with 95% bootstrapped confidence intervals that do not cross zero.

### 3.2 Mediation analyses

PROCESS was used to test whether different types of hope (child hope, parental hope, societal, and denial) mediated the relationships between resolution and stress, with autism awareness and level of support entered as covariates. Due to the high correlation between parental and societal hope, societal hope was dropped from analyses to avoid problems of collinearity (Hayes, 2018)^1^.

The total effect model was significant, *F* = 22.86, (3.68), *p* < 0.001, and explained 50.22% of the variance. The total, *ß* = −0.92, SE = 0.11, 95% CI [−0.70–0.73], *p* < 0.001, and direct effect of resolution on stress were both significant, *ß* = −0.79 SE = 0.15, 95% CI [−0.49–0.63], *p* < 0.001, indicating higher resolution was negatively associated with lower parental stress as predicted.

**Figure 2:**
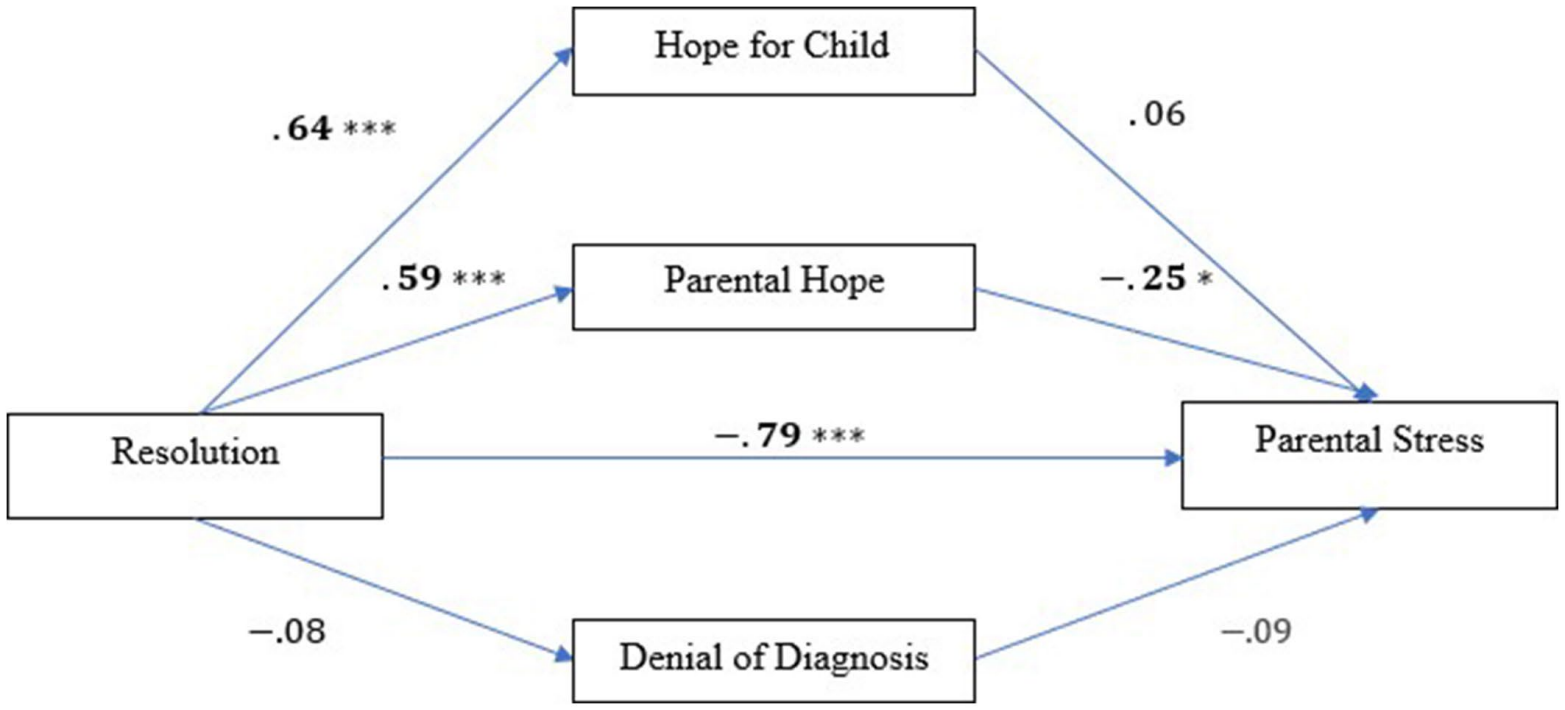
Parallel mediation analyses showing standardized regression coefficients for relationships between resolution, hope (hope for child, parental hope, denial of diagnosis), and parental stress. *p < 0.05, **p < 0.01, ***p <0.001.

There was a significant indirect effect between resolution and stress via parental hope (Figure 2), *ß* = −0.15, SE = 0.07, 95% CI [−0.40, −0.002], indicating that after controlling for variables parental hope mediated the link between resolution and stress. Child-focused hope did not mediate the relationship between resolution and stress as the bootstrapped confidence interval crossed zero, *ß* = 0.04 SE = 0.08, 95% CI [−0.14, 0.19], neither did denial of diagnosis, *ß* = 0.007, SE = 0.02, 95% CI [−0.03, 0.06]. See Table 3 for the overall results of parallel mediation analysis.

**Table 3:** Indirect effect of resolution of diagnosis on parental stress, via child hope, parental hope, and denial of diagnosis.

Predictor	*ß*	*SE*	*t*	*p*	CI95%
DV: child hope	*R* = 0.637, *F* (3.68) = 15.49, *p* < 0.001				
Constant	4.21	1.64	2.56	**0.013**	**[0.93, 7.49]**
Resolution	0.64	0.24	6.52	<0.**001**	**[1.09, 2.05]**
Autism awareness	−0.26	0.34	−2.72	**0.008**	**[−1.60, −0.25]**
Level of support	−0.09	0.21	−0.93	0.355	[−0.61, 0.22]
DV: parental hope	*R* = 0.58, *F* (3.68) = 11.24, *p* = 0.013				
Constant	4.46	1.75	2.55	**0.013**	**[0.98, 7.95]**
Resolution	0.59	0.26	5.64	**<0.001**	**[0.93, 1.96]**
Autism awareness	−0.26	0.36	−2.49	**0.015**	**[−1.62, −0.78]**
Level of support	−0.03	0.22	−0.27	0.791	[−0.50, 0.38]
DV: denial of diagnosis	*R* = 0.56, *F* (3.68) = 10.41, *p* < 0.001				
Constant	11.18	1.67	6.69	**<0.001**	**[7.84, 14.51]**
Resolution	−0.08	0.25	−0.72	0.473	[−0.67, 0.31]
Autism awareness	−0.49	0.35	−4.67	**<0.001**	**[−2.31, −0.93]**
Level of support	−0.28	0.21	−2.73	**0.008**	**[−0.99, −0.15]**
DV: parental stress	*R* = 0.74, *F* (6.65) = 13.23, *p* < 0.001				
Constant	5.30	0.97	5.47	**<0.001**	**[3.37, 7.24]**
Resolution	−0.63	0.15	−5.30	**<0.001**	**[−1.10, −0.50]**
Child hope	0.07	0.07	0.51	0.615	[−0.11, 0.18]
Parental hope	−0.25	0.07	−1.99	**0.047**	**[−0.26, −0.002]**
Denial of diagnosis	−0.09	0.07	−0.89	0.375	[−0.17, 0.06]
Autism awareness	0.13	0.18	1.29	0.202	[−0.13, 0.60]
Level of support	0.01	0.10	0.16	0.874	[−0.18, 0.22]

SE = Standard error. 95% CI = 95% Bias corrected 5,000 bootstrapped confidence intervals. Bold values represent significant relationships, as indicated by 95% confidence intervals that do not cross zero.

## 4 Discussion

We investigated the relationship between resolution to diagnosis and parental stress in parents of autistic children, and whether hope mediated this relationship. Consistent with previous literature (Lord et al., 2008), both correlational and regression analysis supported the hypothesis (H1) that parents with greater resolution to diagnosis reported significantly lower levels of stress. This aligns with the broader literature surrounding significant childhood diagnoses (Sheeran et al., 1997; Sher-Censor and Shahar-Lahav, 2022; Lord et al., 2008), highlighting the importance of resolution as a potential avenue for improved wellbeing of parents. While an important finding, especially in terms of autism research, the role that hope plays in explaining this relationship is an important contribution to the literature.

We positioned hope in line with theoretical perspectives that conceptualize hope as a positive emotion arising in times of uncertainty (e.g., Miceli and Castelfranchi, 2010) for outcomes of significant importance to the individual (Bury et al., 2016). Hope in uncertainty is fitting for parents of children of significant diagnosis, who often report uncertainty regarding their child’s future and their ability to meet the needs of their child (Mulligan et al., 2012; Ogston et al., 2011). Indeed, in this study, parental hopes associated with the child and the child’s future wellbeing, the parents’ ability to support the child, and societal expectations were all strongly endorsed by participants. These hopes were also significantly correlated, suggesting that parents hold multiple hopes for their children simultaneously. Interestingly, while endorsed to a much lower degree, with mean scores below the midpoint of the scale, hopes that the diagnosis was wrong, were also weakly associated with the other hope items. This suggests that although parents can hope that their child can have meaningful life outcomes, and be supported by themselves and society, to a much lesser degree, some parents also hope the diagnosis may be a mistake. However, hope was stronger for positive outcomes representing a resolution to and acceptance of the diagnosis of their child, and particularly for hopes regarding the parent’s ability to meet the needs of their child, which were associated with better wellbeing.

Importantly, as predicted (H3), parental hope was shown to mediate the relationship between resolution to diagnosis and parental stress. Parents resolved to their child’s diagnosis had greater hopes that they could support and would be supported to meet the needs of their child, and this was associated with lower levels of stress. This aligns with research suggesting that resolution in parents has been associated with social support and a perceived sense of competence from the parents (Hotez, 2016; Ekas et al., 2016). After receiving the diagnosis, this shift in adjusting to realistic expectations of the potentially different needs of their child post-diagnosis perhaps provides parents with an understanding of themselves as parents (Brown et al., 2021), perhaps resulting in more realistic hope for themselves as a ‘supporting figure’ in their child’s life (Ogston et al., 2011; Rabba et al., 2019). These realistic expectations may contribute to the reduction in stress, as studies show that parents with a well-supported diagnostic experience and family dynamic feel more at ease compared to parents with poor diagnostic experiences (Abbott et al., 2013; Alsayyari, 2018).

Our results suggest that positive hope for the child did not mediate the relationship between resolution and stress. While positively associated with resolution, when considered in the mediation model, hope for the child was not significantly associated with reduced stress. Compared to parental hope, hopes for the child tended to be more distal, and did not have the direct agency that parental hope has. Although the agency is not necessary for hope to emerge and influence behavior (e.g., Bury et al., 2020), it was not associated with reduced parental stress in this study. Thus, our findings suggest that perhaps parents’ hopes in their ability as a parent is more important and indicative of stress, rather than positive hope for their child. Hence, although parents may have great positive hope regarding their child’s wellbeing and abilities (Mulligan et al., 2012; Ogston et al., 2011; Blake, 2021), it does not have a significant effect on the relationship between resolution and stress.

While hope that a diagnosis was mistaken is not necessarily impossible and thus is not technically denying reality, it certainly seems antithetical to the idea of resolution to diagnosis. That being said, hope in the denial of diagnosis was not associated significantly with either resolution or parental stress in this study. It was also not strongly endorsed by participants (mean scores below the mid-point of the scale). Interestingly, this type of hope was negatively associated with parents who reported a greater level of support needs for their children. While it could be reasonable to hope the diagnosis was wrong for those experiencing a greater impact of a diagnosis on their child’s level of need, Yirmiya et al. (2015) suggest parents may find achieving or maintaining a resolved status easier when increased symptom severity is present. Thus, the reality of greater support needs does not allow parents the luxury to foster thoughts of a mistaken diagnosis. Additionally, more accurate knowledge of autism was significantly negatively correlated with denial of diagnosis, suggesting knowledge may act as a protective factor against unrealistic expectations (Alsayyari, 2018; Heredia-Alvarado, 2017; Rabba et al., 2019).

### 4.1 Theoretical and practical implications

The present study strengthens understanding of the important role resolution to diagnosis has for parent wellbeing, and the importance of hope in this process. These findings suggest that parental hope may be particularly induced in times of uncertainty and likely placed in the possibility of creating more goal-oriented perspectives and formations of hope (Bury et al., 2019). As Rabba et al. (2019) found parents resorted to maintaining hope as a mechanism in managing the uncertainty of receiving a diagnosis, findings support hope, particularly parental hope as a potential protective factor in lessening psychological distress (Lord et al., 2008; Mednick et al., 2007) applied particularly to an autism diagnostic scenario. This aligns with parents expressing the need for support surrounding diagnostic processes and perceived perceptions of social support (Reiff et al., 2017; Rabba et al., 2019, Hotez, 2016). Therefore, parent support and interventions that foster hope in parents’ ability to support their child’s needs may play an important role in parental wellbeing.

### 4.2 Limitations and future directions

While this research positions the role of hope as a potential mechanism explaining some of the relationships between resolution and parental stress, this study was cross-sectional, thus causal conclusions cannot be made. Future studies may consider longitudinal approaches to resolution research to support the role hope plays in resolution. This study finds preliminary evidence suggesting that parental hope has a greater effect on resolution and stress than hope for the child, however, larger more expansive studies would allow for greater exploration of these variables.

Unlike previous studies (Chamak et al., 2011), this study did not find time since diagnosis to be a factor associated with resolution; however, cohort studies may further explore this inconsistency and determine whether the time since diagnosis is potentially causally linked to resolution (Song and Chung, 2010), as longitudinal studies will be better suited to measuring resolution at different stages of the process. Furthermore, we did not account for participants’ experience of the diagnostic process impacting resolution (Abbott et al., 2013). Given that experiences with professionals who take strength-based approaches are associated with better experiences of the diagnostic process (Anderberg and South, 2021), and can have implications for the recipient of that diagnosis (Bury et al., 2022), the nature of the diagnosis could not only have an impact on resolution, but potentially also parental and child hope that feature strength-based concepts.

### 4.3 Conclusion

Our findings provide preliminary evidence that parental hope, which is an important factor arising from resolution, may buffer parents against the effect of parental stress. Given this, hope is focused on practical aspects of parents’ beliefs—specifically their ability to meet their child’s needs. Supporting parents in developing a realistic, strength-based understanding of these needs could be important during the diagnostic process.

## Data Availability

The original contributions presented in the study are included in the article/Supplementary material, further inquiries can be directed to the corresponding author.

## Footnotes

^1^Parental hope was included as it is more frequently cited as a source of hope for parents of autistic children, and it had greater practical outcomes. Running the model separately with societal and parental hope as the sole mediatior (Hayes, 2018) showed both as significant mediators of resolution and parental stress (see Supplementary materials).
