# Supplementary material for: Does hope mediate the relationship between parent’s resolution of their child’s autism diagnosis and parental stress
Source: Front Psychol. 2024 Sep 4;15:1443707. doi: 10.3389/fpsyg.2024.1443707 (PMC11408360; doi:10.3389/fpsyg.2024.1443707)
Supplement: Supplementary file 1 [file Table_1.DOCX]

Supplementary Material

The following four tables reflect the mediation analysis with each of the hope constructs as single mediators.

**Table S1**

*Indirect Effect of Resolution of Diagnosis on Parental Stress, via Child Hope*

| Predictor | *ß* | *SE* | *t* | *p* | CI95% |
| --- | --- | --- | --- | --- | --- |
| DV: Child Hope | *R* = .637, *F*(3,68) = 15.49, p < .001 | | | | |
| Constant | 4.21 | 1.64 | 2.56 | **.013** | **[0.93, 7.49]** |
| Resolution | 0.64 | .24 | 6.52 | <.**001** | **[1.09, 2.05]** |
| Autism Awareness | -0.26 | .34 | -2.72 | **.008** | **[-1.60, -0.25]** |
| Level of Support | -0.09 | .21 | -0.93 | .355 | [-0.61, 0.22] |
| DV: Parental Stress | *R* = .72, *F*(4,67) = 13.28, p < .001 | | | | |
| Constant | 4.56 | .81 | 5.64 | **<.001** | **[2.95, 6.17]** |
| Resolution | -0.65 | .14 | -5. 69 | **<.001** | **[-1.11, -0.53]** |
| Child Hope | -0.13 | .06 | -1.15 | .254 | [-0.18, .05] |
| Autism Awareness | 0.19 | .17 | 2.04 | **.046** | **[0.01, 0.68]** |
| Level of Support | 0.03 | .10 | 0.35 | .728 | [-0.17, 0.23] |
| Total, Direct, and Indirect Effects of Resolution on Parental Stress | | | | | |
| Total Effect | -.92* | .11 | -8.14 | **<.001** | **[-1.15, -0.70]** |
| Direct Effect | -.82* | .14 | -5.69 | **<.001** | **[-1.11, -0.53]** |
| Indirect Effect | -.08 | .07 | - | - | [-0.23, 0.05] |

*Note****.*** SE = Standard error, *unstandardized effect

**Table S2**

*Indirect Effect of Resolution of Diagnosis on Parental Stress, via Parental Hope*

| Predictor | *ß* | *SE* | *t* | *p* | CI95% |
| --- | --- | --- | --- | --- | --- |
| DV: Parental Hope | *R* = .58, *F*(3,68) = 11.24, p = .013 | | | | |
| Constant | 4.46 | 1.75 | 2.55 | **.013** | **[0.98, 7.95]** |
| Resolution | 0.59 | .26 | 5.64 | **<.001** | **[.93, 1.96]** |
| Autism Awareness | -0.26 | .36 | -2.49 | **.015** | **[-1.62, -0.78]** |
| Level of Support | -0.03 | .22 | -0.27 | .791 | [-0.50, 0.38] |
| DV: Parental Stress | *R* = .74, *F*(4,67) = 19.94, p < .001 | | | | |
| Constant | 4.85 | .78 | 6.21 | **<.001** | **[3.29, 6.41]** |
| Resolution | -0.58 | .13 | -5.57 | **<.001** | **[-1.00, -0.48]** |
| Parental Hope | -0.25 | .05 | -2.46 | **.016** | **[-0.23, -0.02]** |
| Autism Awareness | 0.16 | .16 | 1.79 | .079 | [-0.03, 0.61] |
| Level of Support | 0.03 | .09 | 0.42 | .677 | [-0.15, 0.23] |
| Total, Direct, and Indirect Effects of Resolution on Parental Stress | | | | | |
| Total Effect | -.92* | .11 | -8.14 | **<.001** | **[-1.15, -0.70]** |
| Direct Effect | -.74* | .13 | -5.57 | **<.001** | **[-1.00, -0.48]** |
| Indirect Effect | -.14 | .06 | - | - | **[-0.27, -0.02]** |

*Note****.*** SE = Standard error, *unstandardized effect

**Table S3**

*Indirect Effect of Resolution of Diagnosis on Parental Stress, via Societal Hope*

| Predictor | *ß* | *SE* | *t* | *p* | CI95% |
| --- | --- | --- | --- | --- | --- |
| DV: Societal Hope | *R* = .47, *F*(3,68) = 6.58, p = .001 | | | | |
| Constant | 3.91 | 2.19 | 1.78 | **.079** | **[-0.47, 8.29]** |
| Resolution | 0.49 | .32 | 4.40 | **<.001** | **[.77, 2.06]** |
| Autism Awareness | -0.20 | .45 | -1.76 | .082 | [-1.71, 0.11] |
| Level of Support | 0.06 | .28 | 0.52 | .608 | [-0.41, 0.69] |
| DV: Parental Stress | *R* = .73, *F*(4,67) = 19.17, p < .001 | | | | |
| Constant | 4.63 | .77 | 6.00 | **<.001** | **[3.09, 6.17]** |
| Resolution | -0.63 | .13 | -6.36 | **<.001** | **[-1.05, -0.55]** |
| Societal Hope | -0.20 | .04 | -2.13 | **.037** | **[-0.17, -0.01]** |
| Autism Awareness | 0.18 | .16 | 2.08 | **.042** | **[0.01, 0.65]** |
| Level of Support | 0.05 | .10 | 0.62 | .534 | [-0.13, 0.25] |
| Total, Direct, and Indirect Effects of Resolution on Parental Stress | | | | | |
| Total Effect | -.92* | .11 | -8.14 | **<.001** | **[-1.15, -0.70]** |
| Direct Effect | -.80* | .13 | -6.36 | **<.001** | **[-1.04, -0.55]** |
| Indirect Effect | -.10 | .05 | - | - | **[-0.22, -0.01]** |

*Note****.*** SE = Standard error, *unstandardized effect

**Table S4**

*Indirect Effect of Resolution of Diagnosis on Parental Stress, via Denial of Diagnosis*

| Predictor | *ß* | *SE* | *t* | *p* | CI95% |
| --- | --- | --- | --- | --- | --- |
| DV: Denial of Diagnosis | *R* = .56, *F*(3,68) = 10.41, p < .001 | | | | |
| Constant | 11.18 | 1.67 | 6.69 | **<.001** | **[7.84, 14.51]** |
| Resolution | -0.08 | .25 | -0.72 | .473 | [-0.67, 0.31] |
| Autism Awareness | -0.49 | .35 | -4.67 | **<.001** | **[-2.31, -0.93]** |
| Level of Support | -0.28 | .21 | -2.73 | **.008** | **[-0.99, -0.15]** |
| DV: Parental Stress | *R* = .72, *F*(4,67) = 18.07, p < .001 | | | | |
| Constant | 5.23 | .99 | 5.31 | **<.001** | **[3.26, 7.20]** |
| Resolution | -0.74 | .11 | -8.32 | **<.001** | **[-1.16, -0.71]** |
| Denial of Diagnosis | -0.16 | .06 | -1.53 | .132 | [-0.17, 0.03] |
| Autism Awareness | 0.15 | .18 | 1.46 | .150 | [-0.10, 0.63] |
| Level of Support | -0.002 | .10 | -0.02 | .985 | [-0.21, 0.20] |
| Total, Direct, and Indirect Effects of Resolution on Parental Stress | | | | | |
| Total Effect | -.92* | .11 | -8.14 | **<.001** | **[-1.15, -0.70]** |
| Direct Effect | -.94* | .11 | -8.32 | **<.001** | **[-1.16, -0.71]** |
| Indirect Effect | .01 | .02 | - | - | [-0.02, 0.07] |

*Note****.*** SE = Standard error, *unstandardized effect
